# Cloning and heterologous expression of *Plasmodium ovale* dihydrofolate reductase-thymidylate synthase gene

**DOI:** 10.1016/j.parint.2011.12.004

**Published:** 2012-06

**Authors:** Srisuda Tirakarn, Pinpunya Riangrungroj, Palangpon Kongsaeree, Mallika Imwong, Yongyuth Yuthavong, Ubolsree Leartsakulpanich

**Affiliations:** a Department of Chemistry, Mahidol University, Rama 6 Road, Bangkok 10400, Thailand; b Center for Excellence in Protein Structure and Function, Faculty of Science, Mahidol University, Rama 6 Road, Bangkok 10400, Thailand; c National Center for Genetic Engineering and Biotechnology, National Science and Technology Development Agency, 113 Paholyothin Road, Klong 1, Klong Luang, Pathumthani 12120, Thailand; d Department of Clinical Tropical Medicine, Faculty of Tropical Medicine, Mahidol University, Bangkok 10400, Thailand

**Keywords:** DHFR, dihydrofolate reductase, TS, thymidylate synthase, JR, junction region, Pf, *Plasmodium falciparum*, Po, *P. ovale*, Pv, *P. vivax*, Tc, *Trypanosoma cruzi*, CYC, cycloguanil, DTT, dithiothreitol, dUMP, deoxyuridine monophosphate, EDTA, ethylenediaminetetraacetic acid, HCOH, formaldehyde, H_2_folate, 7,8-dihydrofolate, IPTG, iso-propyl-beta-d-thio-galactopyranoside, LC-MS/MS, liquid chromatography-mass spectrometry, MTX, methotrexate, NADPH, nicotinamide adenine dinucleotide phosphate, PVDF, polyvinylidene fluoride, PYR, pyrimethamine, TES, N-Tris(hydroxymethyl)methyl-2-aminoethanesulfonic acid, TMP, trimethoprim, 6-[^3^H] FdUMP, 5-fluoro 2′-deoxyuridine 5′-monophosphate, (6*R*)-CH_2_H_4_folate, 5,10-methylene tetrahydrofolate, MM, minimum media, *Plasmodium ovale*, Dihydrofolate reductase-thymidylate synthase, Antifolates

## Abstract

Plasmodial bifunctional dihydrofolate reductase-thymidylate synthase (DHFR-TS) is a validated antimalarial drug target. In this study, expression of the putative *dhfr-ts* of *Plasmodium ovale* rescued the DHFR chemical knockout and a TS null bacterial strain, demonstrating its DHFR and TS catalytic functions. PoDHFR-TS was expressed in *Escherichia coli* BL21 (DE3) and affinity purified by Methotrexate Sepharose column. Biochemical and enzyme kinetics characterizations indicated that PoDHFR-TS is similar to other plasmodial enzymes, albeit with lower catalytic activity but better tolerance of acidic pH. Importantly, the PoDHFR from Thai isolate EU266602 remains sensitive to the antimalarials pyrimethamine and cycloguanil, in contrast to *P. falciparum* and *P. vivax* isolates where resistance to these drugs is widespread.

## Graphical abstract

**Figure:**
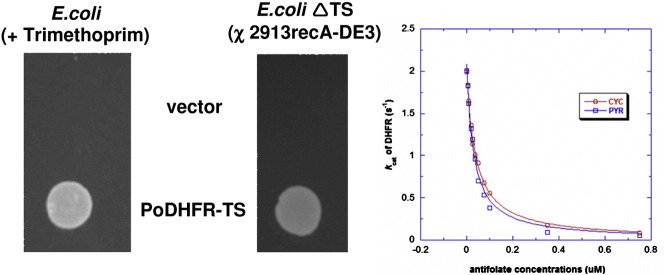

**Highlights:**

► Polymorphism of *P. ovale* dihydrofolate reductase-thymidylate synthase was identified. ► Bacterial complementation assay revealed the function of the putative PoDHFR-TS. ► The protein was expressed and purified. ► Biochemical and kinetic properties including antifolate sensitivity were determined.

## 1 Introduction

Malaria is caused by *Plasmodium* protozoan transmitted by female Anopheles mosquitoes. There are five species that infect human, namely, *P. falciparum* (Pf), *P. vivax* (Pv), *P. malariae* (Pm), *P. ovale* (Po), and *P. knowlesi* (Pk). *P. falciparum* and *P. vivax* are the most prevalent species found in most endemic areas, whereas *P. malariae* and *P. ovale* infections have patchy and limited distribution [1–3]. *P. knowlesi* is a zoonosis parasite recently reported to be the fifth human-infecting malaria species [4]. Mixed infections by these parasite species also are generally observed [5].

Parasite detection by blood smears observed under a light microscope is the most common tool used in the field in order to assess the levels of clinical infection and for epidemiological surveillances. However, differentiation of *Plasmodium* species, especially of *P. ovale*, *P. malariae*, and *P. knowlesi* by light microscopy can be challenging [6]. Indeed, the detection limit of light microscopy has been suggested to be a cause of low incidences reported for malaria cases caused by these species [7]. In addition, information on drug resistance is important in designing strategic plans to control the spread of malaria infection and providing effective treatment for the patients. These data are well established for *P. falciparum* and *P. vivax*, but they have been overlooked for the others. It is well established that a drug deployment policy for one particular malaria parasite species can lead to selection pressure on other *Plasmodium* species, as exemplified by the spillover of antifolate resistant phenotype observed in *P. falciparum* into *P. vivax* [8–10].

Unlike *P. falciparum*, where continuous *in vitro* culture has been routinely established [11], cultures of the other malaria parasite species, including *P. ovale*, have yet to be established. Therefore, determination of *in vitro* drug susceptibility in these parasites cannot be made. However, the putative gene of *P. ovale* dihydrofolate reductase-thymidylate synthase (PoDHFR-TS), a known antifolate target, has recently been identified and sequenced [12]. Here, we report the cloning and heterologous expression of PoDHFR-TS gene from a Thai isolate (EU266602), together with protein purification. Bacterial complementation and biochemical characterization were performed to verify the coding sequence as DHFR-TS. The sensitivity to antifolate antimalaria of the expressed PoDHFR-TS was assessed.

## 2 Materials and methods

### 2.1 Molecular cloning of *P. ovale* dihydrofolate reductase-thymidylate synthase (podhfr-ts) gene

The genomic DNA was prepared from a Thai isolate (EU266602), for which the *P. ovale* infection was confirmed using 2 PCR protocols based on the amplification of species specific small subunit rRNA (SSU rRNA) and linker region (or junction region; jr) of the *dhfr-ts* genes [13,14]. Full-length *podhfr-ts* (1920 bp) was generated by an overlap extension PCR technique [15]. The *dhfr-jr* and *jr-ts* DNA fragments were separately amplified and each fragment contained a 54 bp overlapping flanking sequence in order to mediate homologous recombination that generated the full-length *podhfr-ts* as described below. Unless otherwise indicated, PCR reactions were carried out in a total volume of 50 μL containing 100 ng of DNA template, 125 μM dNTPs, 250 nM each of sense and antisense primers, and 3 U of Pfu polymerase (Promega Corporation, WI, USA).

The sequence coding for *podhfr-jr* was amplified from pGemT-*podhfr* plasmid using primers 5′PoDHFR (ATGGAGGAAGTCTCAGAGGTTTTC) and 3′Po-link (GATGATCCTTTTCCGTGATGAGAAG). PCR thermocycling conditions were as follows: 95 °C for 5 min; 30 cycles of 94 °C for 1 min, 50 °C for 1 min and 72 °C for 2 min; and a final heating at 72 °C for 5 min. The amplicon (863 bp) was purified using GeneJET™ Plasmid Miniprep Kit (Fermentas, Burlington, Canada).

The *pojr-ts* fragment was amplified directly from *P. ovale* genomic DNA using primers 5′Po-link (GCAGGAGCTACCTCTTCCATG) and 3′PoTS (TTAGGCGGCCATATCCATAGTGAT). *Taq* DNA polymerase was used in the PCR with the following thermocycling conditions: 1 cycle of 95 °C for 5 min; 30 cycles of 94 °C 1 min, 55 °C 1 min and 72 °C 1 min; and a final cycle of 72 °C 5 min. The expected *jr-ts* amplicon (1134 bp) was purified from 0.8% agarose using GeneJET™ Gel Extraction Kit (Fermentas, Burlington, Canada). The extracted *pojr-ts* was ligated into T-overhang pTZ57R/T plasmid (Fermentas, Burlington, Canada) and the resulting pTZ57R/T-*pojr-ts* plasmid construct was used as a template to re-amplify *pojr-ts* sequence using conditions as described above, except that Pfu polymerase and an elongation time of 2.5 min were used.

In order to obtain the full-length *podhfr-ts*, 50 ng each of the *podhfr-jr* and *pojr-ts* fragments were combined and PCR was performed without addition of primers using the following thermocycling conditions: 1 cycle of 95 °C for 5 min; 30 cycles of 94 °C for 1 min, 70 °C for 1 min and 72 °C for 2.5 min; and a final step at 72 °C for 5 min. Then 1.0 μL of the PCR solution together with primers 5′NhePo (CACCGCTAGCATGGAGGAAGTCTCAGAGGT; *Nhe* I restriction site as underlined) and 3′PoTS were used in the subsequent PCR with the following thermocycling conditions: 1 cycle of 95 °C for 5 min; 30 cycles of 94 °C for 1 min, 60 °C for 1 min, and 72 °C for 4 min; and a final step at 72 °C for 5 min.

The *podhfr-ts* was digested with *Nhe* I and ligated into pET17b (Invitrogen) vector, which had previously been digested with *Nhe* I and *EcoR* V. The insert sequence was analyzed by 1st BASE (Selangor Darul Ehsan, Malaysia) and Genome Institute (Pathumthani, Thailand).

Site-directed mutagenesis experiments were performed to correct any mutations occurring. The reaction contained 50 ng template, 125 μM dNTPs, 250 nM sense and antisense primers, and 3 U of Pfu polymerase. Similar PCR conditions as described above were employed, except that the condition for annealing and elongation was 61 °C for 1 min and 72 °C for 11 min respectively. Sense and antisense primers used to correct the mutations are listed in Table 1, which shows the corrected nucleotides (underlined).

### 2.2 Complementation assay

For DHFR activity test, *E. coli* BL21 (DE3) was transformed with pET17bPoDHFR-TS and plated on LB agar containing 100 μg/ml ampicillin. A single colony of transformed cell was cultured in 5 mL of LB broth supplemented with 100 μg/ml ampicillin at 37 °C until OD_600_ reached 0.8. Cells (5 μL) were spotted on minimum media (MM) agar plate supplemented with 100 μg/ml ampicillin in either the presence or absence of 4 μM trimethoprim (TMP), and incubated at 37 °C overnight. Cells transformed with pET17b were used as a negative control.

For TS activity test, a Thy¯ *E. coli* strain χ 2913recA-DE3 (Δ *thyA572, recA56*) [16] was used. Similar experiment to that described for DHFR activity test was performed, except that MM agar plate contained 100 μg/ml ampicillin in the presence or absence of 50 μg/ml thymidine.

### 2.3 PoDHFR-TS enzyme expression and purification

*E. coli* BL21 (DE3) harboring pET17bPoDHFR-TS plasmid was used for expression of PoDHFR-TS. Cells were grown in SOB medium supplemented with 100 μg/ml ampicillin at 37 °C until OD_600_ reached 1.0, when expression of PoDHFR-TS was induced by supplementation with 0.4 mM IPTG. Cells were grown at 16 °C for 20 h, collected by centrifugation at 10,000 *g* for 10 min and lysed using a French press (Thermo Dynamics Ltd., Nova Scotia, Canada) at 69 MPa in buffer A (20 mM potassium phosphate pH 7.0, 50 mM KCl, 0.1 mM EDTA, 10 mM DTT, and 20% (v/v) glycerol). Cell lysate was centrifuged at 27,000 *g* for 1 h, and PoDHFR-TS in the supernatant was purified using MTX-affinity chromatography as previously described [17]. Briefly, the supernatant was loaded onto MTX-Sepharose pre-equilibrated with buffer A and the nonspecific binding proteins were washed by buffer A containing 1 M KCl. PoDHFR-TS was eluted by buffer B (50 mM TES pH 8.0, 0.1 mM EDTA, 10 mM DTT, 50 mM KCl, 20% (v/v) glycerol, and 4 mM H_2_folate).

Fractions containing DHFR activity was further purified by passage over a Sephacryl S-200 HR column (2.5 × 90 cm), using buffer A at a flow rate of 0.2 mL/min. Another approach used for PoDHFR-TS purification was Q-Sepharose anion-exchange chromatography (3 × 5 cm column) using a linear salt gradient from 0.05 to 1 M NaCl in buffer A (100 mL total volume). SDS-PAGE was used to demonstrate protein expression and purity.

### 2.4 PoDHFR-TS enzyme identification

Protein bands observed on Coomassie blue-stained SDS-PAGE were subjected to an in-gel tryptic digestion, and identified by LC-MS/MS (Genome Institute, Pathumthani, Thailand). All MS/MS spectra were searched against database using SEQUEST algorithm by filtering of cross-correlation score (X*corr*) versus charge state (+ 1 ≥ 1.5,+2 ≥ 2.0,+3 ≥ 2.5) with protein probability of a minimum of 1.00 × 10^− 3^) and Sp (Primary Score) of greater than 500.

For *N*-terminal sequence analysis, protein bands on SDS-PAGE were transferred onto PVDF membrane (Millipore, Massachusetts, USA) and subsequently sequenced by Edman degradation method (AltaBioscience, Birmingham, UK).

### 2.5 Kinetic characterization of DHFR and TS activities

DHFR enzyme activity was measured spectrophotometrically at 340 nm for the decrease of NADPH substrate and the activity was calculated using a molar absorptivity (ε_340_) of 12,300 M^− 1^ cm^− 1^ [18]. One unit of DHFR activity is defined as the amount of the enzyme that catalyzes the reduction of 1 μmol of substrate per minute at 25 °C. The assay solution (1 mL) contained 50 mM TES pH 7.0, 75 mM β-mercaptoethanol, 1 mM EDTA, 100 μM H_2_folate, 100 μM NADPH, 1 mg/ml BSA and enzyme. *K*_m_ values for H_2_folate and NADPH were conducted at a range of 1–100 μM for one substrate while keeping fixed the concentration of the other at 100 μM. All reactions were initiated with 0.005 enzyme unit and performed in a Hewlett Packard UV–VIS spectrophotometer (HP8453). Data were fitted to a Michaelis–Menten equation using a non-linear least-square fitting module of KaleidaGraph™ software (Synergy Software, Pennsylvania, USA).

TS enzyme activity was measured spectrophotometrically by monitoring at 25 °C the increase in absorbance at 340 nm due to the formation of H_2_folate, performed with an approximately 0.006 enzyme unit in TS buffer (50 mM TES pH 7.4, 25 mM MgCl_2_, 6.5 mM HCOH, 1 mM EDTA and 75 mM β-mercaptoethanol), 150 μM of (6*R*)-CH_2_H_4_folate (Merck Eprova AG, Switzerland), and 125 μM dUMP (to initiate reaction). Enzyme activity was calculated using a molar extinction coefficient (ε_340_) of 6,400 M^− 1^ cm^− 1^ [19]. One unit of TS activity is defined as the amount of the enzyme that catalyzes the formation of 1 μmol of product per minute. *K*_m_ values for (6*R*)-CH_2_H_4_folate and dUMP were performed using a saturation concentration of one substrate (150 μM for (6*R*)-CH_2_H_4_folate and 125 μM for dUMP) and varying the concentration of the other (2–150 μM for (6*R*)-CH_2_H_4_folate and 1–125 μM for dUMP), and data were analyzed as described for DHFR.

### 2.6 6-[^3^H] FdUMP thymidylate synthase labeling activity

TS labeling by 6-[^3^H] FdUMP was performed as previously described [20–22]. In brief, 2 μg of purified PoDHFR-TS were incubated with 5 μL of 3 mM (6*R*)-CH_2_H_4_folate, 0.25 μL of 66 μM 6-[^3^H] FdUMP (15 Ci/mmol, Moravek Biochemicals, California, USA) in TS buffer (20 μL final volume) at 25 °C for 1 h. The reaction was stopped by adding SDS dye solution and boiling for 10 min. The protein complex was analyzed by 12.5% SDS-PAGE. The Coomassie-blue dye stained gel was soaked in autoradiography enhancer (NEN™, Dupont) for 1 h at 25 °C with gently shaking, dried at 80 °C, and exposed to Hyperfilm (Amersham Hyperfilm ECL™, GE Healthcare Life Science) at − 80 °C for one day. The procedure was performed with *Trypanosoma cruzi* DHFR-TS as positive control and with PvDHFR as a negative control.

### 2.7 Effect of pH on reaction catalysis

The pH dependences for DHFR and TS activities were monitored as described in Section 2.5 but using polybuffer in the pH range of 4–9.5 at 25 °C. For assay of DHFR activity the polybuffer was composed of 50 mM acetic acid, 50 mM MES, 100 mM Tris-Cl, and 10 mM β-mercaptoethanol, and for TS activity the polybuffer buffer was supplemented with 25 mM MgCl_2_, 6.5 mM HCOH, and 1 mM EDTA. Catalytic constant was plotted as a function of pH.

### 2.8 Inhibition of DHFR by antifolates

Inhibition of DHFR activity by antifolate compounds [pyrimethamine (PYR) and cycloguanil (CYC)] were determined in 1 mL of DHFR assay solution containing 100 μM NADPH and H_2_folate, together with 5 nM–1 μM inhibitors. Reaction was initiated by adding 0.01 enzyme unit. Inhibition constant (*K*_i_) was calculated from a non-linear least-square fit of data using KaleidaGraph™ software. The type of inhibition by antifolate compounds was analyzed using DHFR assay solution containing 100 μM NADPH with varying (2.5 μM–12.5 μM) H_2_folate concentrations. Data were analyzed using a Lineweaver–Burk plot of the reciprocal values of initial velocity and concentrations of H_2_folate.

## 3 Results and discussion

### 3.1 Analysis of PoDHFR-TS sequence

**Fig. 1:**
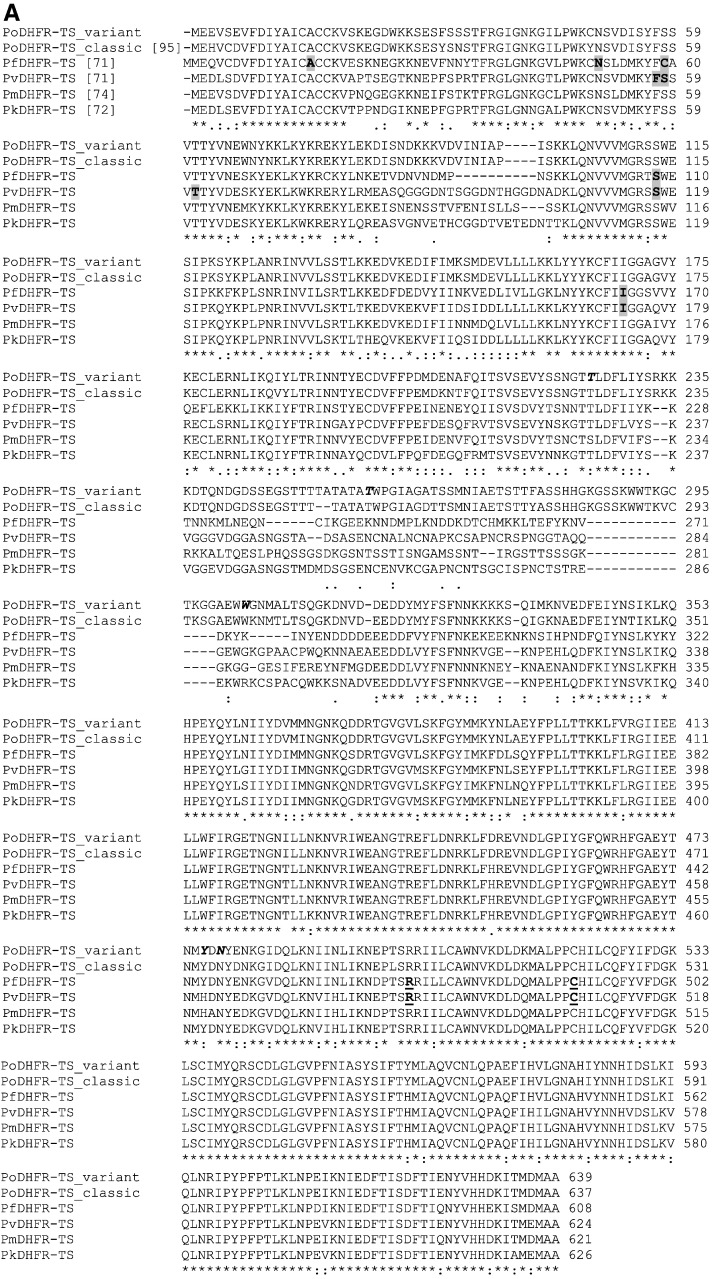

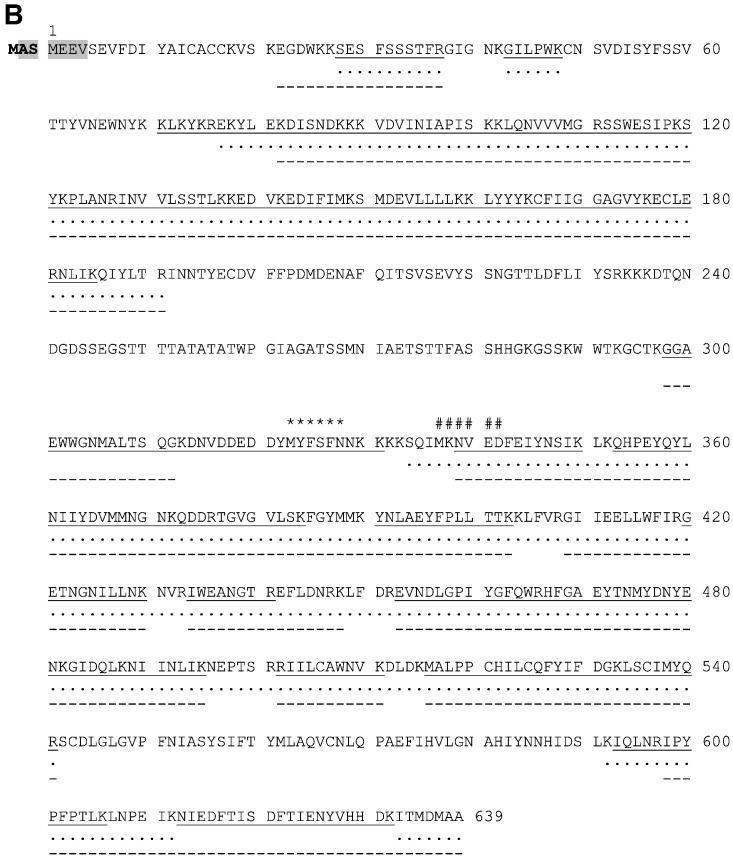
Sequence analyses. (A) Multiple amino acid sequence alignment of PoDHFR-TS variant (EU266602), PoDHFR-TS classic (EU266606), PfDHFR-TS (J03028), PvDHFR-TS (EU478859), PmDHFR-TS (AY846633), and PkDHFR-TS (XM002258192). Numbers in brackets indicate percent homology to PoDHFR-TS variant EU266602. Amino acids A16, N51, C59, S108, and I164 in PfDHFR-TS and F57, S58, T61, S117, I173 in PvDHFR-TS are highlighted in bold. Mutations at these positions have been reported to be associated with antifolate resistance. Residues known to be important for TS activity are shown as underlined bold letters. T225, T258, W303, Y476, and N478 — for which the errors were found in the encoded nucleotides during the cloning process — are denoted in italic with bold letters. (*) amino acid positions with identical residue, (:) amino acid positions with conserved substitution amino acid, (.) amino acid positions with semi-conserved substitution amino acid, and (non-marked) amino acid positions with non-conserved substitution amino acid. (B) The results by LC-MS/MS of tryptic digested fragments of 74 (solid line), 35 (dotted line), and 32 (dash line) kDa proteins matched to DHFR-TS of *P. ovale*. Highlighted amino acids are *N*-terminal sequences obtained by Edman *N*-terminal sequencing of 74, 35, and 32 kDa proteins. Asterisk (*) and hash (#) symbols represent sequences of 35 and 32 kDa protein respectively, identified by Edman *N*-terminal sequencing. The first three amino acids (bold letters) are extra residues generated from cloning of *podhfr-ts* into *Nhe* I site of pET17b.

A recent study by multi-locus sequence analysis identified 2 nonrecombining sympatric forms of *P. ovale*, classic and variant types, or *P. ovale curtisi* and *P. ovale wallikeri* respectively [12]. At present, 18 *podhfr-ts* sequences derived from field isolates of different geographic regions have been reported [12]. Sequence comparisons reveal dimorphic patterns in PoDHFR-TS (Table 2), and the variant type contains 2 amino acids more than that of the classic type (639 versus 637 residues) in the junction region (Fig. 1A). *P. ovale* variant type is mostly observed for Thai isolates (8 out of 12), while the classic type is from Sao Tome (2 out of 6) and Guinea-Bissau (4 out of 6) (Table 2). It is noted that none of the parasites from Thai isolates belong to the classic type. Examples of dimorphic patterns observed in DHFR domain are demonstrated — F/Y31, S/N33, C/Y49, I/V187, D/E204, E/K207, and A/T209 (Table 2). These polymorphisms are not equivalent to those associated with antifolate resistance in PfDHFR-TS and PvDHFR-TS. Thus, it is unlikely that the PoDHFR-TS enzyme would be resistant to antifolates, unless these polymorphisms significantly affect the binding characteristics of the active site. Point mutations in *Plasmodium* DHFR responsible for antifolate resistance have been well characterized for *P. falciparum* (A16V, N51I, C59R, S108N, and I164L) and *P. vivax* (F57L, S58R, T61M, S117N, and I173L) [23,24]. These residues in variant and classic PoDHFRs are identical — A15, N50, F57, S58, T61, S113, and I169 — which are similar to the wild-type sequence of other *Plasmodium* DHFRs (Table 2). The conserved R470 and C490 in PfDHFR-TS are important for TS activity [16], for which these residues are equivalent to R501 and C521 of PoDHFR-TS variant or R499 and C519 for the classic type (Fig. 1A). Using *P. ovale* strain EU266602, a variant type, as the reference sequence, a number of sequence variations were identified among different isolates such as E3Q/H, V4L, E6D, I39V, Y56C, I96M, Y164H , C166R, C178Y, I184M, and I212V. These variations were confirmed as the sequences of each isolate were derived from at least 2 colonies after cloning.

### 3.2 Construction of *podhfr-ts* expression vector

Initial attempts to PCR amplify the open reading frame (ORF) of *podhfr-ts* from genomic DNA resulted in the production of partial gene sequences. Eventually, the full-length *podhfr-ts* was successfully amplified using an overlap extension PCR-based method (Fig. S). In this approach, *podhfr* and *pots* with flanking homologous sequence were synthesized first (Fig. S; PCR 2), and the amplicons were then used as templates and primers for sequence extension in the following PCR (Fig. S; PCR 3). Another PCR was required in order to obtain sufficient amounts of DNA for further cloning step (Fig. S; PCR 4). A major amplicon of 1920 bp was finally obtained (Fig. S; PCR 4). Reasons for the unsuccessful amplification of the full-length gene by direct PCR amplification from genomic DNA include primers annealing at non specific sites, and presence of DNA secondary structures due to the high AT content (67%) thereby hindering polymerase processivity.

Upon ligation at *Nhe* I and *EcoR* V sites, 3 amino acids, namely, methionine, alanine, and serine, are introduced upstream of the initiation methionine of PoDHFR-TS. Five missense mutations (T225I, ACA → ATA; T258S, ACA → TCA; W303R, TGG → AGG; Y476H, TAT → CAT; and N478D, AAT → GAT) and one missing nucleotide (A708) of K236 were found from the first expression construct generated. Amino acids T225, T258, W303, Y476, and N478 are conserved in all 18 isolates, in which the sequences were reported [12]. The errors observed were likely to be due to the use of multiple steps of PCR and of *Taq* DNA polymerase, a non proofreading DNA polymerase [25]. The sequence was corrected by site-directed mutagenesis and the final expression construct, pET17bPoDHFR-TS, encodes 642 amino acids, which share 65–75, 19–32, and 90% identities for the DHFR, JR, and TS domains, respectively, with other human plasmodial species (Fig. 1A).

### 3.3 Complementation assay

**Fig. 2:**
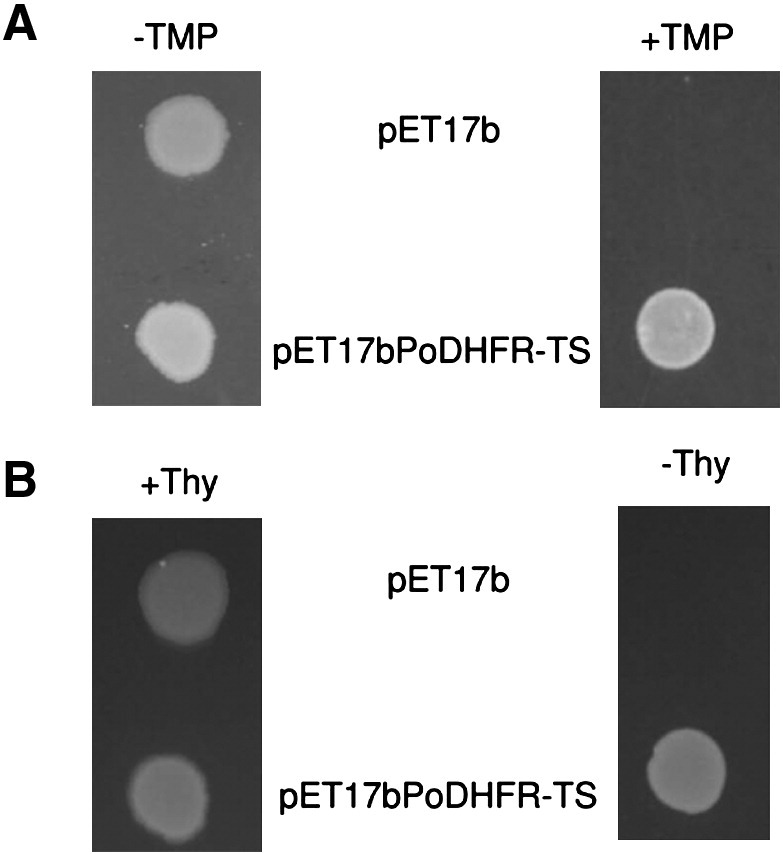
Bacterial complementation assays to demonstrate DHFR and TS functions of PoDHFR-TS coded sequence. (A) *E. coli* BL21 (DE3) transformed with either pET17b or pET17bPoDHFR-TS was grown on MM in the absence or presence of 4 μM TMP. (B) *E. coli* χ 2913recA-DE3 carrying pET17b or pET17bPoDHFR-TS was cultured on MM with and without 50 μg/ml thymidine. MM used in A and B also contained 100 μg/ml ampicillin.

Bacterial complementation assays were carried out to provide preliminary evidence for DHFR and TS functions of the heterologously expressed protein. DHFR activity was investigated through its ability to complement bacterial cell activity in the presence of TMP, a specific bacterial DHFR inhibitor. *E. coli* transfected with pET17bPoDHFR-TS could grow on MM plate containing 4 μM TMP, whereas cells carrying an empty plasmid was suppressed (Fig. 2A). A similar strategy was employed for TS activity determination, using Thy¯ *E. coli* strain χ 2913recA-DE3. Such cells carrying pET17bPoDHFR-TS were able to grow in the absence of thymidine supplementation, as was required by untransfected control cells (Fig. 2B).

Since at present it is not possible to maintain *P. ovale* in continuous culture, this bacterial complementation assay provides a useful means to evaluate novel compounds with potential inhibitory properties directed against *P. ovale* DHFR and TS, as well as evaluating mutant enzymes from field isolates and constructed in the laboratory. A similar approach was recently used for screening antifolates for PvDHFR [26].

### 3.4 Enzyme expression and purification

**Fig. 3:**
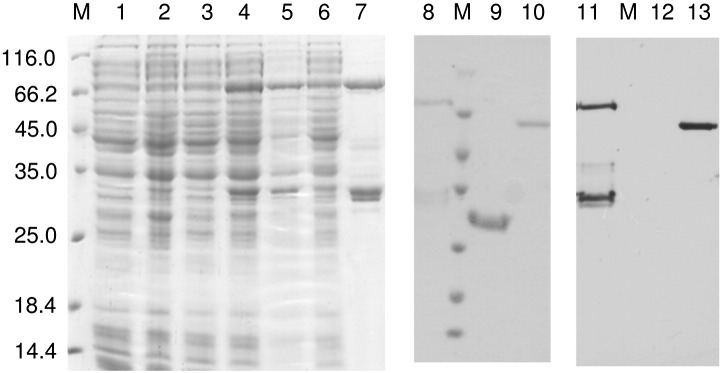
Coomassie blue-stained SDS-PAGE and X-ray film exposed to [^3^H] FdUMP labeling of recombinant PoDHFR-TS. Lane M, molecular weight markers; (1) uninduced cells carrying pET17b; (2) induced cells carrying pET17b; (3) uninduced cells carrying PoDHFR-TS gene; (4) induced cell carrying PoDHFR-TS gene; (5) insoluble fraction of (4); (6) soluble fraction of (4); (7) purified PoDHFR-TS after MTX-affinity chromatography; (8–10) purified PoDHFR-TS, PvDHFR and TcDHFR-TS; (11–13) autoradiographs of (8–10) following [^3^H] FdUMP labeling.

Heterologous expression of recombinant PoDHFR-TS in *E. coli* BL21 (DE3) induced with 0.4 mM IPTG at 16 °C for 20 h was relatively low (~ 1%) as assessed by SDS-PAGE analysis (Fig. 3). Attempts to increase yield by varying host cell types and IPTG concentrations were unsuccessful (data not shown). The expressed PoDHFR-TS protein was purified using MTX-affinity chromatography, yielding 12 mg of enzyme, with a specific activity of 1.7 μmol/min/mg, from 1 L culture. Upon the analysis by SDS-PAGE, in addition to the expected protein band of 74 kDa, proteins of 35 and 32 kDa also were observed, accounting for 50% of the total protein in the gel (Fig. 3). Further purification by gel filtration and anion-exchange chromatographies failed to remove these smaller proteins (data not shown). These results are similar to those previously reported for the purification of heterologous expressed PfDHFR-TS, in which it was concluded that the phenomenon is the consequence of either protein degradation or premature termination of protein translation as evidenced from protein *N*-terminal sequencing results [22]. Although the smaller fragments may be from any region of PoDHFR-TS, as these fragments were co-purified by MTX-affinity chromatography, they were assumed to contain the DHFR domain. Further experiments to express PoDHFR-TS containing *C*-terminus tagged with either single or double hexa-histidine sequence to facilitate purification of the full-length PoDHFR-TS from the truncated fragments were equally fruitless (data not shown). The specific activities of the DHFR domain measured in the crude extracts derived from the expression of these latter constructs were significantly lower than that from pET17bPoDHFR-TS (data not shown), suggesting interference of DHFR activity by the presence of *C*-terminal histidine tags.

To determine the identities of 74, 35, and 32 kDa proteins, these bands were separately subjected to tryptic digestion and the tryptic peptides were analyzed by LC-MS/MS. The MS/MS spectra of these fragments were searched against an in-house created *Plasmodium* database using Biowork^TM^ 3.3 software (Thermo Scientific, USA). The tryptic peptides of these three samples corresponded to PoDHFR-TS with the best score among *Plasmodium* DHFR-TS. The amino acid sequences of tryptic fragments of all three samples corresponded well to the *in silico* digested peptides in DHFR, JR, and TS domains (Fig. 1B).

To confirm the presence of PoDHFR-TS, Edman *N*-terminal sequence analysis was conducted. After methionine deblocking, the first 6 amino acid sequence obtained from 74 kDa protein was ASMEEV, perfectly matching the *N*-terminus of the recombinant PoDHFR-TS, which has an extra AS *N*-terminal sequence derived from cloning into *Nhe* I site of pET17b. Thus, the 74 kDa protein is the bona-fide full-length PoDHFR-TS, based on mass, MS and *N*-terminal sequencing data.

However, *N*-terminal sequencing of the 32- and 35-kDa bands did not produce unequivocal sequencing results, but indicated the presence of two polypeptides with *N*-terminal sequences ASMEEV (amino acids 1–4) and MKNVED (amino acids 337–342) present in the 32 kDa band, and ASMEEV and MYFSFN (amino acids 323–328) in the 35 kDa band. These polypeptide fragments are presumably due to a chymotrypsin-like proteolytic activity [27].

TS activity in the expressed PoDHFR-TS was determined by taking the advantage of the ability of FdUMP, a TS suicide inhibitor, to form a covalent adduct with TS. Autoradiography revealed that [^3^H] FdUMP labeled 32, 35 and 74 kDa bands (Fig. 3), in line with the data from LC-MS/MS and *N*-terminal sequencing.

It can be concluded that the bifunctional PoDHFR-TS had been obtained and had co-purified with truncated fragments containing both DHFR and TS domains. These results also suggest the existence of tight interactions between these domains. Mutations in the proteolytic cleavage region may prevent the production of such fragments during protein expression, with the caveat that the mutant protein does not affect enzyme activity.

### 3.5 Effect of pH on DHFR and TS activities

**Fig. 4:**
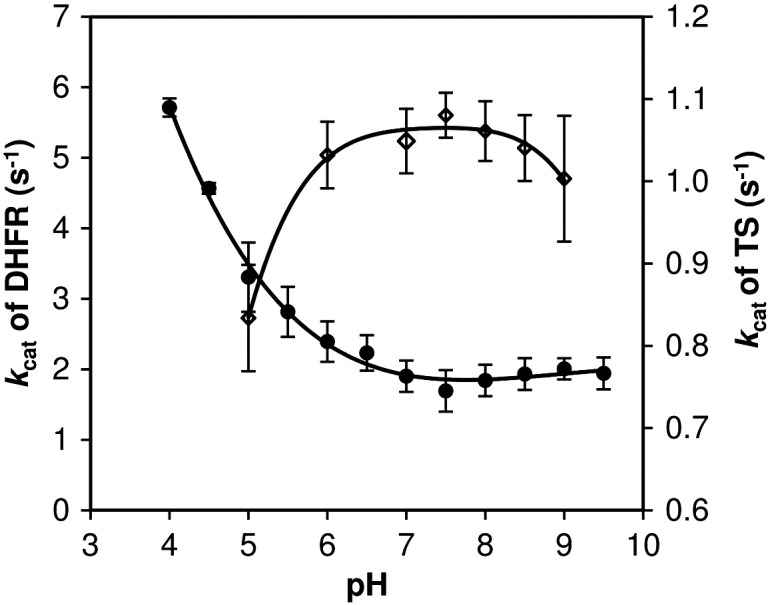
Effect of pH on DHFR (filled circles) and TS (open diamonds) activities of *P. ovale*. The catalytic constants were determined for reactions performed over pH range of 4–9.5. Each data point is the average value from three experiments.

The relationship between steady state catalytic constant and pH for DHFR and TS demonstrated that the PoDHFR domain of the bifunctional PoDHFR-TS was active in both the acid and neutral ranges but the activity decreased at higher pH (Fig. 4). The optimum pH could not be determined owing to the instability of H_2_folate at pH < 4. Although *Plasmodium* DHFRs are conserved in sequence, the pH profile obtained for PoDHFR is different from those of PfDHFR and PvDHFR, which exhibit maximum activity around pH 7 [24,28]. However, a number of vertebrate DHFRs have higher activity in acidic pH [29], similar to our observation for PoDHFR. On the other hand, TS activity was similar at pH 7–9.

### 3.6 DHFR and TS kinetic properties

Steady state kinetic parameters for PoDHFR-TS as well as the inhibition constants for antifolates (PYR and CYC) were determined and are summarized in Table 3. The apparent *K*_m_ values for NADPH and H_2_folate are similar to those of other *Plasmodium* DHFRs. It is worth noting that the DHFR domain of *P. ovale* enzyme is the least catalytically active, as its *k*_cat_ is 9 and 2.5 folds lower than that of the wild-type PfDHFR and PvDHFR respectively. The TS domain of PoDHFR-TS also has half of the activity of that of other *Plasmodium* species.

**Fig. 5:**
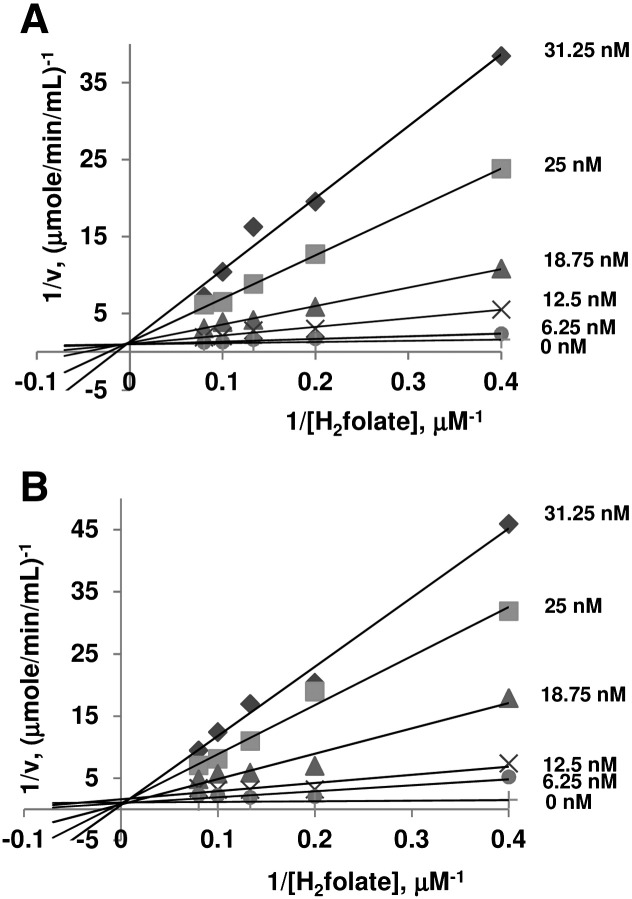
Double reciprocal plots of inhibition of DHFR domain of PoDHFR-TS by (A) PYR (0–31.25 nM) and (B) CYC (0–31.25 nM). Assays were performed in the presence of NADPH (100 μM) and H_2_folate (2.5–12.5 μM). The final concentrations of inhibitors are indicated on each line.

Standard kinetics double reciprocal plots revealed that PYR and CYC inhibited H_2_folate binding to PoDHFR domain in a competitive fashion (Fig. 5). The antimalarial antifolates, PYR and CYC, inhibited PoDHFR at nanomolar concentrations (Table 3), similar to other wild-type *Plasmodium* enzymes. Thus, *P. ovale* would appear to be sensitive to antifolates, but constant monitoring of resistance in the field is clearly warranted.

In summary, this study reports the cloning, sequencing and heterologous expression of *podhfr-ts*. Biochemical characterization of the purified recombinant bifunctional PoDHFR-TS showed that it was similar in kinetic properties with other wild-type *Plasmodium* species, but with anomaly pH activity profile, and that the DHFR domain was sensitive to the antifolates, PYR and CYC. Due to the availability of *podhfr-ts*, this paves the way for further structure–function relationship studies and drug susceptibility test of site-directed mutant enzymes, towards the goal of rational drug design directed against *P. ovale*.

The following are the supplementary materials related to this article.

**Fig. S:**
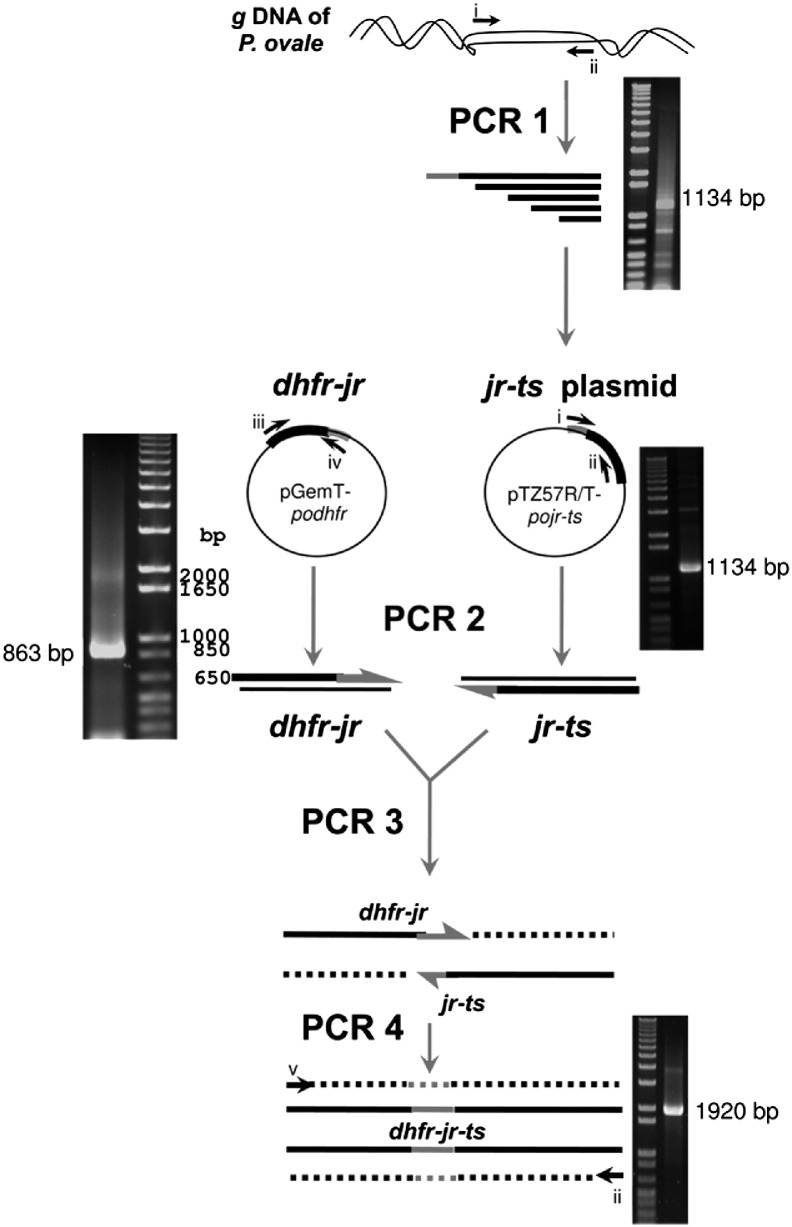
Schematic representation of generation of full-length *podhfr-ts* using overlap extension PCR technique and analysis of amplicons. Four PCR reactions were required to generate the complete sequence: (1) *pojr-ts* (1134 bp) was PCR amplified from *P. ovale* DNA and ligated into pTZ57R/T; (2) *podhfr-jr* (863 bp) and *pojr-ts* (1134 bp) was acquired using pGEMT-*podhfr* and pTZ57R/T-*pojr-ts* as template respectively; (3) and (4) *podhfr-ts* (1920 bp) was obtained by 2-steps PCR overlap extension, in which *dhfr-jr* and *jr-ts* contain overlapping sequences that are complementary for extension shown by half arrow heads indicating the direction in which each fragment can act as primers for DNA polymerase. Dashed lines mark newly synthesized sequence or strand. Horizontal arrows represent primers used in PCR reactions: i, ii, iii, iv, and v are 5′Po-link, 3′PoTS, 5′PoDHFR, 3′Po-link, and 5′NhePo, respectively.

## Figures and Tables

**Table 1:** Oligonucleotides used to perform site-directed mutagenesis to correct the sequence of cloned *podhfr-ts.*

Corrected mutation	Sense (F) and antisense (R) primers used
I225T	F; GTAGTAACGGAACCACATTAGACTTTCTAATTTA
	R; TAAATTAGAAAGTCTAATGTGGTTCCGTTACTAC
236K	F; TTACAGTAGGAAAAAAAAAGACACACAAAATG
	R; CATTTTGTGTGTCTTTTTTTTTCCTACTGTAA
S258T	F; GCTACAGCTACAGCGACATGGCCAGGAATAG
	R; CTATTCCTGGCCATGTCGCTGTAGCTGTAGC
R303W	F; GGAGCAGAATGGTGGGGAAATATGGCT
	R; AGCCATATTTCCCCACCATTCTGCTCC
H476Y, D478N	F; GCTGAATATACAAATATGTATGATAATTATGAAAATAAAGGAATTG
	R; CAATTCCTTTATTTTCATAATTATCATACATATTTGTATATTCAGC

Corrected nucleotides are underlined.

**Table 2:** Sequence analysis of DHFR domain among five *Plasmodium* species as well as the variant and classic dimorphic types of *P. ovale* from different regions. Accession numbers J03028, EU478859, AY846633, and XM002258192 belong to PfDHFR-TS (Pf), PvDHFR-TS (Pv), PmDHFR-TS (Pm), and PkDHFR-TS (Pk). Amino acid positions in PfDHFR-TS (16, 51, 59, 108, and 164) and PvDHFR-TS (57, 58, 61, 117, and 173) implicated in antifolate resistance are bolded. The symbol (.) represents the same amino acid as that presence in *P. ovale* strain EU 266602 (bolded letter). ND = no data available.

Accession no.	Origin	Amino acid number in DHFR domain																													
J03028 (Pf)	FCR3, Lab strain		4	5	6	7	14	**16**	32	34	40	50	**51**	57	58	**59**	62	71	91	**108**	113	159	161	**164**	173	179	182	199	202	204	207
			Q	V	C	D	I	A/V	F	N	L	C	N/I	Y	F	C/R	T	E	D	S/N,T	P	Y	C	C I/L	F	I	I	E	E	E	I
EU478859 (Pv)	India		3	4	5	6	13	15	31	33	39	49	50	56	**57**	**58**	**61**	70	100	**117**	122	168	170	**173**	182	188	191	208	211	213	216
			D	L	S	D	I/L	A	F	P	L	C	N	Y	F/L	S/R	T/M	E	G	S	P	Y	C	I	C	I	I	E	E	Q	V
AY846633 (Pm)	Thailand		3	4	5	6	13	15	31	33	39	49	50	56	57	58	61	70	97	114	119	165	167	170	179	185	188	205	208	210	213
			E	V	S	D	I	A	F	T	L	S	N	Y	F	S	T	K	S	S	P	Y	C	I	C	I	I	E	E	V	I
XM002258192 (Pk)	Malaysia		3	4	5	6	13	15	31	33	39	49	50	56	57	58	61	70	100	117	122	168	170	173	182	188	191	208	208	213	216
			D	L	S	E	I	A	F	P	L	C	N	Y	F	S	T	E	T	S	P	Y	C	I	C	I	I	Q	E	Q	M
		**Type**	3	4	5	6	13	15	31	33	39	49	50	56	57	58	61	70	96	113	118	164	166	169	178	184	187	204	207	209	212
**EU266602**	Thailand	Variant	E	V	S	E	I	A	F	S	I	C	N	Y	F	S	T	K	I	S	P	Y	C	I	C	I	I	D	E	A	I
GQ250091	PNG	Variant	Q	L	.	D	.	.	.	.	.	.	.	.	.	.	.	.	.	.	.	.	.	.	.	.	.	.	.	.	.
EU266604	Benin	Variant	Q	L	.	D	.	.	.	.	.	.	.	.	.	.	.	.	.	.	.	.	.	.	.	.	.	M	.	.	.
EU266605	Sao Tome	Variant	Q	L	.	D	.	.	.	.	V	.	.	C	.	.	.	.	.	.	.	.	.	.	.	.	.	.	.	.	.
EU266612	Thailand	Variant	ND	ND	ND	ND	.	.	.	.	.	.	.	.	.	.	.	.	.	.	.	.	.	.	.	.	.	.	.	.	.
EU266613	Thailand	Variant	ND	ND	ND	ND	.	.	.	.	.	.	.	.	.	.	.	.	.	.	.	H	.	.	.	.	.	.	.	.	.
EU266614	Thailand	Variant	ND	ND	ND	ND	.	.	.	.	.	.	.	.	.	.	.	.	.	.	.	.	.	.	.	.	.	.	.	.	.
EU266615	Thailand	Variant	ND	ND	ND	ND	.	.	.	.	.	.	.	.	.	.	.	.		.	.	.		.	.	.	.	.	.	.	.
EU266616	Thailand	Variant	ND	ND	ND	ND	.	.	.	.	.	.	.	.	.	.	.	.	.	.	.	.	.	.	Y	.	.	.	.	.	.
EU266617	Thailand	Variant	ND	ND	ND	ND	.	.	.	.	.	.	.	.	.	.	.	.	.	.	.	.	.	.	.	.	.	.	.	T	.
EU266618	Thailand	Variant	ND	ND	ND	ND	.	.	.	.	.	.	.	.	.	.	.	.	.	.	.	.	.	.	.	.	.	.	.	.	.
GQ250090		Variant	.	L	C	.	.	.	.	.	.	.	.	.	.	.	.	.	.	.	.	.	.	.	.	.	.	.	.	.	.
EU266606	Sao Tome	Classic	H	.	C	D	.	.	Y	N	.	Y	.	.	.	.	.	N	.	.	.	.	.	.	.	.	V	E	K	T	.
EU266607	Sao Tome	Classic	ND	ND	ND	ND	.	.	Y	N	.	Y	.	.	.	.	.	N	.	.	L	.	.	.	.	.	V	E	K	T	.
EU266608	Guinea-Bissau	Classic	ND	ND	ND	ND	.	.	Y	N	.	Y	.	.	.	.	.	N	.	.	.	.	.	.	.	.	V	E	K	T	.
EU266609	Guinea-Bissau	Classic	ND	ND	ND	ND	.	.	Y	N		Y	.	.	.	.	.	D	M	.	.	.	.	.	.	.	V	E	K	T	.
EU266610	Guinea-Bissau	Classic	ND	ND	ND	ND	.	.	Y	N	.	Y	.	.	.	.	.	N	.	.	.	.	R	.	.	.	V	G	K	T	.
EU266611	Guinea-Bissau	Classic	ND	ND	ND	ND	.	.	Y	N	.	Y	.	.	.	.	.	N	.	.	.	.	.	.	.	.	V	E	K	T	V

**Table 3:** Kinetic parameters of purified PoDHFR-TS in comparison with PfDHFR-TS and PvDHFR-TS.

*Plasmodium* enzyme	DHFR activity			TS activity			DHFR inhibition	
	*K*_m_ [H_2_folate] (μM)	*K*_*m*_ [NADPH] (μM)	*k*_cat_ (s^− 1^)	*K*_m_ [CH_2_H_4_folate] (μM)	*K*_*m*_ [dUMP] (μM)	*k*_cat_ (s^− 1^)	*K*_i_ [PYR] (nM)	*K*_i_ [CYC] (nM)
PoDHFR-TS	2.42±0.25	3.71±0.51	8.5±0.6	5.92±0.77	1.44±0.16	0.50±0.07	0.67±0.08	0.96±0.15
PfDHFR-TS	1.0±0.1^a^	4.2±0.2^a^	80^a^	2.12±0.26^b^	1.66±0.14^b^	1.13^b^	0.2±0.02^c^	0.3±0.00^c^
PvDHFR-TS	1.6±0.1	1.1±0.2	21.2	2.77±0.71^d^	3.72±0.67^d^	1.20^d^	0.21±0.03^e^	0.31±0.04

a–e; data from [28], [16], [30], [31], and [26], respectively.
